# Defining the relationship between phylogeny, clinical manifestation, and phenotype for *Trichophyton mentagrophytes/interdigitale* complex; a literature review and taxonomic recommendations

**DOI:** 10.1093/mmy/myad042

**Published:** 2023-04-17

**Authors:** Michaela Švarcová, Tomáš Větrovský, Miroslav Kolařík, Vit Hubka

**Affiliations:** Department of Genetics and Microbiology, Faculty of Science, Charles University, , Prague, Czech Republic; Laboratory of Environmental Microbiology, Institute of Microbiology, Czech Academy of Sciences, Prague, Czech Republic; Laboratory of Fungal Genetics and Metabolism, Institute of Microbiology, Czech Academy of Sciences, Prague, Czech Republic; Department of Botany, Faculty of Science, Charles University, Prague, Czech Republic; Laboratory of Fungal Genetics and Metabolism, Institute of Microbiology, Czech Academy of Sciences, Prague, Czech Republic; Department of Botany, Faculty of Science, Charles University, Prague, Czech Republic

**Keywords:** anthropophilic dermatophytes, dermatophytosis, multigene phylogeny, skin infections, zoophilic dermatophytes

## Abstract

This study looked for correlations between molecular identification, clinical manifestation, and morphology for *Trichophyton interdigitale* and *Trichophyton mentagrophytes*. For this purpose, a total of 110 isolates were obtained from Czech patients with various clinical manifestations of dermatophytosis. Phenotypic characters were analyzed, and the strains were characterized using multilocus sequence typing. Among the 12 measured/scored phenotypic features, statistically significant differences were found only in growth rates at 37 °C and in the production of spiral hyphae, but none of these features is diagnostic. Correlations were found between *T. interdigitale* and higher age of patients and between clinical manifestations such as tinea pedis or onychomychosis. The MLST approach showed that internal transcribed spacer (ITS) genotyping of *T. mentagrophytes* isolates has limited practical benefits because of extensive gene flow between sublineages. Based on our results and previous studies, there are few taxonomic arguments for preserving both species names. The species show a lack of monophyly and unique morphology. On the other hand, some genotypes are associated with predominant clinical manifestations and sources of infections, which keep those names alive. This practice is questionable because the use of both names confuses identification, leading to difficulty in comparing epidemiological studies. The current identification method using ITS genotyping is ambiguous for some isolates and is not user-friendly. Additionally, identification tools such as matrix-assisted laser desorption/ionization time-of-flight mass spectrometry fail to distinguish these species. To avoid further confusion and to simplify identification in practice, we recommend using the name *T. mentagrophytes* for the entire complex. When clear differentiation of populations corresponding to *T. interdigitale* and *Trichophyton indotineae* is possible based on molecular data, we recommend optionally using a variety rank: *T. mentagrophytes* var. *interdigitale* and *T. mentagrophytes* var. *indotineae*.

## Introduction

Dermatophytes are pathogenic fungi that cause superficial mycoses in vertebrates.^1^ The prevalence of dermatophytosis in the human population is estimated to be 20–25% on a worldwide scale, and *Trichophyton rubrum, Trichophyton interdigitale* and *Trichophyton mentagrophytes* are among the most common pathogens, although the prevalence of specific mycoses can vary widely.^2^ The differentiation of *T. mentagrophytes* and *T. interdigitale* is considered epidemiologically and clinically relevant by some authors because the two species cause infections with different clinical presentations and because internal transcribed spacer (ITS) genotyping enables the recognition of potential sources of infection and terbinafine resistance.^3,4^

The taxonomic status of *T. mentagrophytes* and *T. interdigitale* has been the subject of much controversy. In 1999, the taxonomic classification of these pathogens changed due to neotypifications by Gräser et al.^5^ The neotype selection of *T. mentagrophytes* significantly changed the meaning of this well-known name in practice, as the neotype was related or even identical to *Trichophyton quinckeanum*.^6^ Due to this change, *T. mentagrophytes* became rare in clinical practice. The concept of “anthropophilic and zoophilic strains” of *T. interdigitale* was used during a transitional period,^4,7^ and, consequently, the majority of isolates that had been previously identified as *T. mentagrophytes* were identified as zoophilic strains of *T. interdigitale*. The selection of the neotype of *T. mentagrophytes* was disputed by some authors,^6,8,9^ and in light of new arguments, an alternative neotype was designated by de Hoog et al.^10^ Although the validity of this neotype may be the subject of future nomenclature debate, we follow this designation here.

*Trichophyton mentagrophytes* is considered a zoophilic species with a host spectrum including rodents, cats, dogs, and, less commonly, other animals, such as ruminants and horses.^11^ When transmitted to humans, infection usually manifests as an inflammatory tinea of glabrous skin (tinea corporis, faciei, and barbae) and less frequently as tinea capitis.^7,12^ Classically, the typical appearance included colonies in shades of beige and yellow cream with a granular/powdery colony texture, numerous globose to subglobose microconidia, and the presence of spiral hyphae and usually also macroconidia. Mating of isolates with opposite mating types leads to the production of a sexual state corresponding to former *Arthroderma vanbreuseghemii*. In contrast, *T. interdigitale* is an anthropophilic species that is considered to be a clonal offshoot derived from the sexual zoophilic lineage of *T. mentagrophytes*. This clonal lineage contains only isolates of one mating type idiomorph and has lost the ability to mate with *T. mentagrophytes*. Typical morphology described in past literature included white and cottony colonies, clavate microconidia, and no or rare macroconidia and spiral hyphae.^1^,^13–15^ In clinical practice, the strains of *T. interdigitale* are almost exclusively associated with onychomycosis and tinea pedis in humans and are absent in animals.^4,7^

In total, 28 ITS genotypes have been identified among *T. mentagrophytes*/*T. interdigitale* isolates, five of which are considered to be *T. interdigitale*.^3,16^ Some genotypes have supposedly specific geographic distributions or are more frequently associated with certain clinical manifestations.^3,17^ A species name *Trichophyton indotineae* was recently proposed for *T. mentagrophytes* isolates of ITS genotype VIII, which was a predominant cause of the Indian epidemic of superficial mycoses and which frequently shows terbinafine resistance.^18,19^ Although this species has been validly described, its definition relies on clinical criteria and ITS genotyping and shatters an unstable taxonomy for *T. mentagrophytes/T. interdigitale*. It was originally described as an anthropophilic species but it was shown that isolates with identical genotype also circulate between animals.^20,21^

The morphological differentiation of *T. mentagrophytes/T. interdigitale* in practice is difficult or impossible given the diversity of transitional growth forms and overall poor correlation between molecular species identification and phenotype.^4,7,12,22^ Additionally, in the majority of phylogenetic studies based on one or several genetic loci, *T. mentagrophytes* and *T. interdigitale* are resolved as paraphyletic or even polyphyletic.^3,12^,^23–25^ This fact further complicates routine diagnostics and raises questions about the taxonomic legitimacy of these species.

In this study, we assessed whether the maintenance of multiple species names has a relevant taxonomic basis and whether it is beneficial for clinical practice. We examined correlations between species identity, clinical differentiation, and morphology for 110 *T. interdigitale/T. mentagrophytes* strains obtained from Czech patients with various clinical manifestations of dermatophytosis. Multigene phylogenetic analysis served as a basis for species identification and it should also verify or refute the monophyly of species and uncover incongruence between single-gene phylogenies.

## Materials and methods

### Material examined

A total of 110 strains morphologically identified as *T. interdigitale/T. mentagrophytes* were obtained from Czech patients with various manifestations of dermatophytosis, including onychomycosis, tinea pedis, and tinea corporis (including the tinea faciei subtype). The strains were prospectively collected from several clinical institutions in the Czech Republic between 2014 and 2017. Detailed information on isolation sources is given in Supplementary Table S1. Selected isolates with unique multilocus genotypes were deposited into the Culture Collection of Fungi (CCF), Department of Botany, Charles University, Prague, Czech Republic.

The phylogenetic analysis was enriched by the ex-type or representative strains of *Trichophyton* species described in the past but considered synonyms of *T. interdigitale* and *T. mentagrophytes*. These cultures were obtained from the CBS culture collection housed at the Westerdijk Institute (Utrecht, The Netherlands). The alternative ex-neotype strain of *T. mentagrophytes* IHEM 4268^10^ was obtained from BCCM/IHEM Fungi Collection (Belgian Coordinated Collections of Microorganisms, Fungi Collection: Human and Animal Health, Sciensano, Brussels, Belgium), and reference strains of *T. indotineae*^18^ were kindly provided by Prof. Rui Kano (Teikyo University Institute of Medical Mycology, Tokyo, Japan).

### Molecular studies

DNA was extracted from 7-day-old colonies using a Quick-DNA Miniprep Kit (Zymo Research, Irvin, CA) according to the manufacturer's protocol. A NanoDrop 1000 spectrophotometer was used to evaluate the quality of the extracted DNA.

Three genetic markers were used for the molecular characterization of the studied strains. The ITS rDNA region (ITS1-5.8S-ITS2 cluster) was amplified using primers ITS1F and ITS4,^26,27^ the partial *tubb* gene encoding β-tubulin was amplified with primers Bt2a and Bt2b,^28^ and the *tef1α* gene encoding translation elongation factor 1-α was amplified with primers EF-DermF and EF-DermR.^29^ The PCR protocol by Hubka et al.^30^ was followed. Polymerase chain reaction product purification was performed using ExoSAP-IT™ PCR Product Cleanup Reagent (Thermo Fisher Scientific, Waltham, MA) in accordance with the manufacturer's protocol. Automated sequencing was conducted by BIOCEV Sequencing Service (Prague, Czech Republic). Unique DNA sequences were deposited into the GenBank database, and the accession numbers are listed in Table 1.

**Table 1.** Accession numbers for strains with unique multilocus genotypes and reference strains used for phylogenetic reconstruction

		GenBank/ENA/DDBJ accession numbers			
Lineage/species	Culture collection numbers^1^	ITS	ITS genotype^2^	*tubb*	*tef1α*
*Trichophyton interdigitale* lineage (Czech patients)	CCF 6575 = CLIS 3036/16	OM283519	Unique ITS genotype A	OM314975	OM568763
	CCF 6577 = DMF 3680/14	OM283526	Ti II	OM314982	OM568767
	CCF 6583 = L307/15	OM283527	Unique ITS genotype B	OM314983	OM568775
	CLIS 9064/16	OM283515	Ti II	OM314970	OM568771
	CCF 6682 = ME 517/15	OQ076537	Ti II	OQ095324	OQ095322
*Trichophyton mentagrophytes* lineage (Czech patients)	CCF 6572 = CLIS 1062/17	OM283516	Tm III*	OM314972	OM568760
	CCF 6573 = CLIS 1182/16	OM283517	Tm IV	OM314973	OM568761
	CCF 6574 = CLIS 2548/16	OM283518	Tm III*	OM314974	OM568762
	CCF 6576 = CLIS 5116/16	OM283520	Tm III*	OM314976	OM568764
	CCF 6578 = CLIS 7172/15	OM283521	Tm III*	OM314977	OM568765
	CCF 6579 = CLIS 985/17	OM283522	Tm III*	OM314978	OM568766
	CCF 6580 = D303/15	OM283523	Tm VII	OM314979	OM568768
	CCF 6581 = D488/15	OM283524	Unique ITS genotype C	OM314980	OM568773
	CCF 6582 = D749/16	OM283525	Unique ITS genotype C	OM314981	OM568774
	CCF 6584 = ME 742/15	OM283528	Tm III*	OM314984	OM568776
	CCF 6585 = ME 940/15	OM283529	Unique ITS genotype D	OM314985	OM568777
	SK 1007/16	OQ076538	Unique ITS genotype E	OQ095325	OQ095323
*Trichophyton indotineae* lineage (Czech patients)	CCF 6599 = SK 3253/16	OM283512	Tm VIII	OM314968	OM568780
**Reference strains**					
*T. interdigitale*	CBS 428.63	OM283536		OM314992	OM568754
*T. verrucosum* var. *autotrophicum* (*T. interdigitale*)	CBS 100378	OM283530		OM314986	OM568748
*T. candelabrum* (*T. interdigitale*)	CBS 647.73	OM283540		OM314996	OM568758
*T. rotundum* (*T. interdigitale*)	CBS 287.30	OM283531		OM314987	OM568749
*T. batonrougei* (*T. interdigitale*)	CBS 425.63	OM283535		OM314991	OM568753
*T. krajdenii* (*T. interdigitale*)	CBS 475.93	OM283538		OM314994	OM568756
*T. mentagrophytes* var. *nodulare* (*T. interdigitale*)	CBS 429.63	OM283537		OM314993	OM568755
*T. mentagrophytes* var. *goetzii* (*T. interdigitale*)	CBS 392.68	OM283534		OM314990	OM568752
*T. mentagrophytes*	IHEM 4268	OM283542		OM314998	OM568769
*T. radicosum* (*T. mentagrophytes*)	CBS 304.38	OM283532		OM314988	OM568750
*Arthroderma vanbreuseghemii* (*T. mentagrophytes*)	CBS 646.73	OM283539		OM314995	OM568757
*T. indotineae*	CCF 6597 = NUBS 19006	OM283543		OM314999	OM568778
*T. indotineae*	CCF 6598 = NUBS 19007	OM283544		OM315000	OM568779
**Outgroups**					
*T. papillosum* (*T. schoenleinii*)	CBS 347.55	OM283533		OM314989	OM568751
*T. langeronii* (*T. schoenleinii*)	CBS 764.84	OM283541		OM314997	OM568759
*Trichophyton quinckeanum*	CLIS 7581/19	OM283513		OM314969	OM568770
*Trichophyton quinckeanum*	ME 1374/18	OM283514		OM314971	OM568772

1 Culture collection acronyms: CBS, Westerdijk Fungal Biodiversity Institute (formerly Centraalbureau voor Schimmelcultures), Utrecht, The Netherlands; CCF, Culture Collection of Fungi, Department of Botany of Charles University, Prague, Czech Republic; IHEM (BCCM/IHEM), Belgian Coordinated Collections of Micro-organisms, Fungi Collection: Human and Animal Health, Sciensano, Brussels, Belgium; NUBS, Nihon University College of Bioresource Sciences, Fujisawa, Kanagawa, Japan; D, DMF, CLIS, L, ME, SK—personal designation of strains (no permanent preservation).

2 ITS genotypes are numbered according to the Klinger et al.^3^ and Uhrlaß et al.^16^

### Phylogenetic analysis and genotype network

DNA sequence alignments of the ITS, *tubb* and *tef1α* loci were performed using the FFT-NS-i option implemented in the MAFFT online service.^31^ The alignments were trimmed and analyzed using maximum likelihood (ML) method. Alignment partitioning schemes and substitution models were determined using PartitionFinder 2 based on the Bayesian information criterion with settings allowing introns, exons, and segments of the ITS region to be independent datasets.^32^ The optimal partitioning schemes for the analyzed dataset are listed in Supplementary Table S2. The phylogenetic tree based on the ML method was constructed with IQ-TREE v. 2.1.2 with nodal support determined by nonparametric bootstrapping with 1000 replicates.^33^ The tree was rooted with a lineage comprising *T. quinckeanum* and *T. schoenleinii* isolates (*T. simii* complex). The graphical outputs of the phylogenetic tree were generated in iTOL (Interactive Tree Of Life).^34^ PopART software was used to create haplotype networks using the TCS method.^35,36^ Alignments are available in the DRYAD digital repository: https://doi.org/10.5061/dryad.6wwpzgn3d.

### MAT locus determination

Identification of MAT locus idiomorphs was performed by amplification of partial MAT gene sequences with previously developed primers. The alpha box domain encoding the *MAT1-1-1* gene was amplified with the primers TmMAT3S and TmMAT3R.^37^ The high mobility group (HMG) domain encoding *MAT1-2-1* was amplified with the primers TmHMG2S and TmHMG2R.^37^ The PCR protocol of Čmoková et al. was followed.^38^ Amplicons were visualized via an electrophoretogram (1% agarose gel with 0.5 μg/mL ethidium bromide). Several samples from each MAT gene were selected for DNA sequencing and confirmation of specificity.

### Phenotypic studies

Isolates were incubated on Sabouraud's glucose agar (SGA) at 25 °C and 37 °C. The sizes of the colonies were measured after one week of cultivation, and macromorphological characteristics were evaluated after two weeks of cultivation for all clinical strains from Czech patients. The colors of the colony centre obverse and reverse were scored according to Kornerup and Wanscher and sorted into groups based on their similarity.^39^ This resulted in two groups for the colony obverse (white, or white with yellow/orange tint) and three groups for the colony reverse (peach yellow, brownish orange, and deep brown) (Supplementary Table S3). The textures of colonies were classified into 3 groups: cottony, velvety, and granular.

The presence or absence of spiral hyphae and macroconidia was evaluated in 2–3-week-old colonies. Dimensions of microconidia were recorded in 50 selected strains (20 *T. interdigitale*, 30 *T. mentagrophytes*); at least 35 measurements per strain.

### Statistical analysis

Correlations were assessed between species identification (*T. interdigitale* vs. *T. mentagrophytes*) and phenotypic markers (growth rates at 25 °C and 37 °C, colony texture, colony color, presence of macroconidia, length of microconidia, width of microconidia, and length/width of microconidia) or clinical characteristics (clinical manifestation—tinea corporis, tinea pedis, and onychomycosis; age of patient, and gender of patient). In the case of patient age or dimensions of microconidia, quantitative values were used directly for analysis, and the remaining characters were scored into categories as described above. A list of all characters and their scoring is given in Supplementary Table S3. Correlations were performed in R software using the generalized least squares model with the package nlme (linear and nonlinear mixed effects models).^40,41^

## Results

### Phylogeny and mating-type gene distribution

Sequences of ITS, *tubb* and *tef1-α*, from 129 isolates belonging to the *T. interdigitale/T. mentagrophytes* complex, were generated for phylogenetic analysis. This dataset comprised 110 strains from Czech patients, 11 CBS/IHEM reference strains from *T. interdigitale/T. mentagrophytes* lineages, 2 reference strains from *T. indotineae* (CCF 6597 and CCF 6598), and an outgroup, i.e., a clade containing 6 isolates from *T. quinckeanum/T. schoenleinii* (*T. simii* complex). The final combined alignment consisted of 1644 characters, of which 74 were variables and 68 were parsimony informative. Alignment characteristics together with partitioning schemes and substitution models are presented in Supplementary Table S2. The accession numbers for the strains with unique multilocus DNA haplotypes are shown in Table 1.

**Figure 1.**
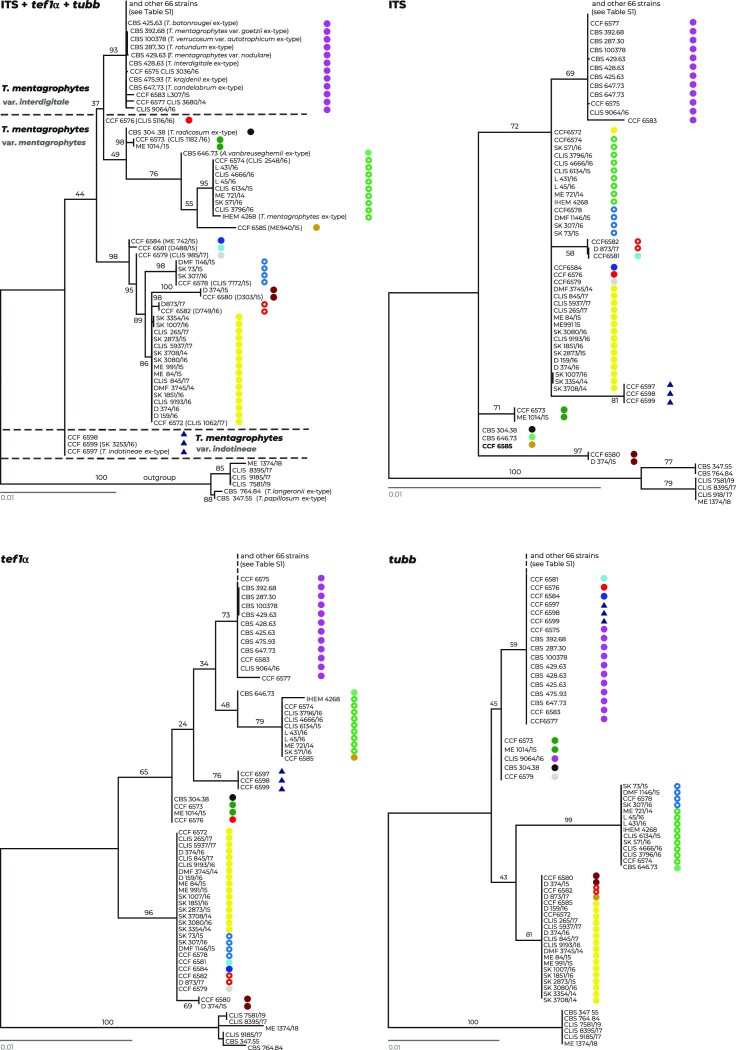
A comparison of the combined phylogeny based on three loci, ITS rDNA, *tef1-α*, and *tubb* (upper left tree), with single-gene phylogenies based on individual loci. All trees represent the best scoring maximum likelihood trees constructed in IQ-Tree. The alignment characteristics, partitioning scheme, and substitution models are listed in Supplementary Table S2. Strains with similar haplotypes/from the same clade are designated with colored shapes to highlight their different or similar position and clustering across trees. The clade containing isolates belonging to the *T. simii* complex is used as the outgroup.

**Figure 2.**
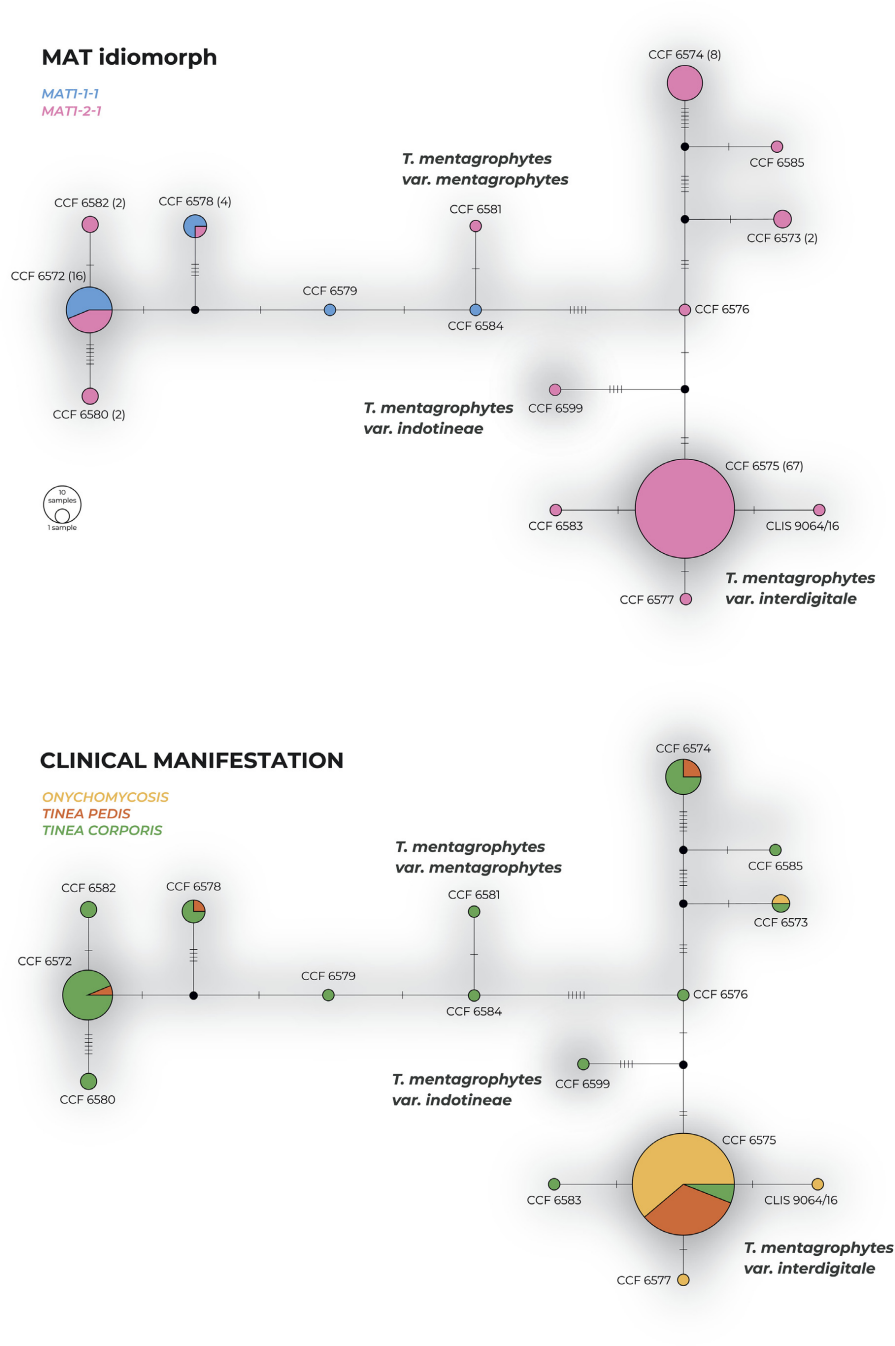
Haplotype network of Czech clinical isolates from the *Trichophyton mentagrophytes* complex examined in this study. The network is based on multilocus data (ITS rDNA, *tubb*, and *tef1-α* loci). Haplotypes are represented by circles with sizes corresponding to the number of isolates. Dashes on connecting lines indicate substitutions (indels excluded) between connected haplotypes. The upper network shows a distribution of MAT gene idiomorphs among isolates having the same haplotype, while the lower network shows a distribution of clinical manifestations.

The ML analysis based on three genes resolved the strains into several clades that were usually poorly supported (Fig. 1), probably due to significant incongruences between single-gene phylogenies. Almost no intraspecies variability was present in the clade containing the ex-type strain of *T. interdigitale* CBS 428.63. This clade comprised 70 isolates from Czech patients and 8 reference strains, including the ex-type strains of *T. verrucosum* var. *autotrophicum* CBS 100378, *T. candelabrum* CBS 647.73, *T. rotundum* CBS 287.30, *T. batonrougei* CBS 425.63, *T. krajdenii* CBS 475.93, *T. mentagrophytes var. goetzii* CBS 392.68, and a representative strain of *T. mentagrophytes* var. *nodulare* CBS 429.63. All 78 strains are considered *T. interdigitale* for the purposes of further analyses. Among Czech strains, only CCF 6583, CCF 6577, and CLIS 9064/16 exhibited unique multilocus genotypes with unique substitutions as shown in the haplotype network (Fig. 2). There were three ITS genotypes detected among Czech strains in the *T. interdigitale* lineage. ITS genotype II sensu Uhrlaβ et al.^16^ was predominant, and two strains with unique genotypes were absent from this numbering system (those designated A–B in Table 1) - strain CCF 6575 had insertion in the ITS1 region and strain CCF 6583 had unique substitution in the ITS2 region. All strains in the *T. interdigitale* lineage exhibited only the *MAT1-2-1* idiomorph of the mating type gene, and the Czech clinical isolates were mostly isolated from onychomycosis and tinea pedis (Fig. 2).

Three strains of *T. indotineae*, ex-type strain CCF 6597, ex-paratype strain CCF 6598, and one Czech clinical strain CCF 6599, were resolved (without significant statistical support) in a separate clade basal to *T. interdigitale* and *T. mentagrophytes* isolates (Fig. 1). In a significant portion of trees, in contrast to best scoring ML tree shown in Fig. 1, *T. indotineae* was resolved as sister to *T. interdigitale* or located inside *T. mentagrophytes* lineage (trees not shown). There was no genetic variability detected among them, and all strains exhibited only the *MAT1-2-1* idiomorph of the mating type gene.

The remaining strains were considered *T. mentagrophytes* and clustered into several poorly supported clades that were paraphyletic to the *T. interdigitale* clade (Fig. 1). These clades contained an alternative ex-neotype strain of *T. mentagrophytes* IHEM 4268 designated by de Hoog et al.^10^ and ex-type strains of *T. radicosum* CBS 304.38 and *Arthroderma vanbreuseghemii* CBS 646.73. There were 11 multilocus genotypes with unique substitutions among the 39 strains from Czech patients (Fig. 2). Six ITS genotypes were detected among the Czech strains of *T. mentagrophytes* (excluding *T. indotineae*). ITS genotype III* sensu Uhrlaβ et al.^16^ was predominant, followed by genotypes IV and VII. In addition, strains with unique genotypes absent from the numbering system were designated C–E in Table 1. The strains exhibited a 25:14 ratio of mating type gene idiomorphs *MAT1-2-1*: *MAT1-1-1*. The strains were predominantly isolated from patients with tinea corporis (Fig. 2).

The single-gene phylogenies were not congruent (Fig. 1), and neither *T. interdigitale, T. mentagrophytes*, or *T. indotineae* was resolved as monophyletic. *Trichophyton indotineae* was conspecific with *T. interdigitale* in the tree based on the *tubb* gene, while in the trees based on ITS and *tef1α* genes, it was surrounded by *T. mentagrophytes* strains. The position of many *T. mentagrophytes* strains was unstable and changed between trees, suggesting extensive gene flow/recombination. To a lesser extent, there are signs of gene flow between strains of *T. mentagrophytes* and *T. interdigitale*. This can be demonstrated by the variable position of four strains, namely, CLIS 9064/16, CCF 6584, CCF 6581, and CCF 6576, which are based on different genes belonging to either *T. mentagrophytes* or *T. interdigitale* (Fig. 1). These data illustrate that no species is phylogenetically well defined and that the boundaries between these putative species are poorly established and permeable.

### Association of phenotypic and clinical features with species identity

**Figure 3.**
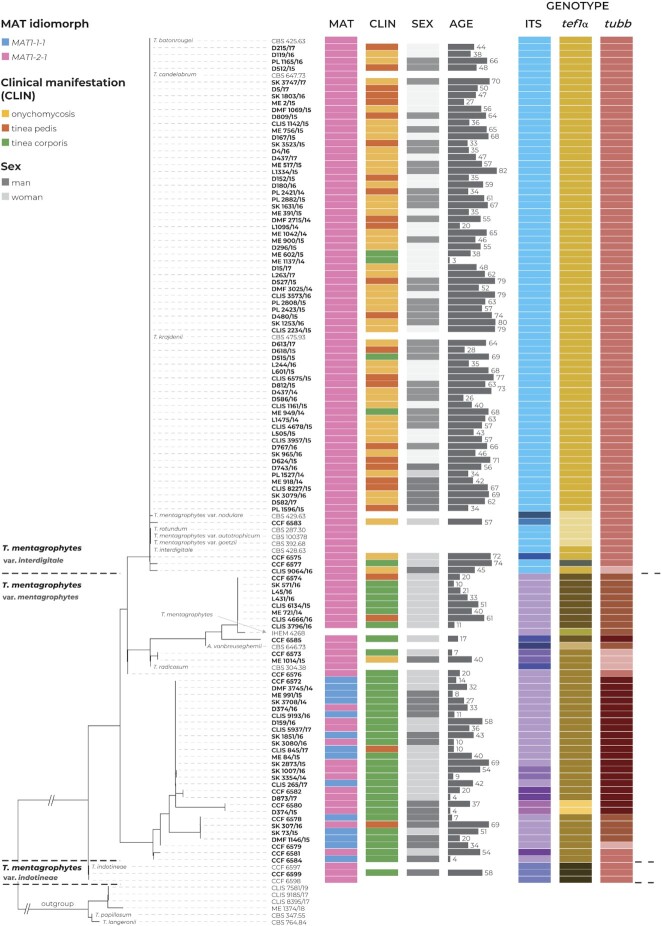
Multilocus phylogenetic tree of the *Trichophyton mentagrophytes* complex inferred with the maximum likelihood method based on ITS rDNA, *tubb*, and *tef1-α* loci (alignment characteristics, partitioning scheme, and substitution models are listed in Supplementary Table S2). A total of 129 isolates were included in the phylogeny, and additional information, such as mating type gene idiomorph (MAT), clinical manifestation (CLIN), sex and age of the patients, and assignment to genotype separately for every locus, is displayed. The clade containing isolates belonging to the *T. simii* complex was used as the outgroup.

The Czech clinical strains were assigned to *T. interdigitale* (*n* = 70) and *T. mentagrophytes* (*n* = 39) according to phylogenetic analysis based on three genes. The clinical data associated with these strains are plotted on the phylogeny in Fig. 3. Correlation analyses were performed on Czech isolates only and are summarized in Table 2. The Czech isolate of *T. indotineae* was excluded from statistical analysis.

**Table 2.** Statistical analysis of differences in distributions of phenotypic features and clinical attributes between isolates of *T. interdigitale* and *T. mentagrophytes*^1^

Character categories	Examined characters	*T. interdigitale*	*T. mentagrophytes*	*P* value
Growth parameters (SGA)	25 °C, 7 d (mm): range (mean)	15–35 (24)	16–33 (24)	0.83
	37 °C, 7 d (mm): range (mean)	6–24 (15)	8–32 (22)	**<0.01**
Colony characteristics (SGA)	Colour of colony obverse—white : yellow or orange tint (see Supplementary Table S3)	46 : 20	22 : 15	0.12
	Colour of colony reverse—peach yellow : brownish orange : deep brown (Supplementary Table S3)	16 : 27 : 23	0 : 10 : 27	0.60
	Colony texture—cottony : velvety : granular	26 : 31 : 7	7 : 8 : 22	0.12
Micromorphology (2–3-week-old cultures, SGA)	Spiral hyphae—present : absent	14 : 6	18 : 12	**0.01**
	Macroconidia—present : absent	2 : 18	7 : 23	0.08
	Length of microconidia: mean ± sd	4.1 ± 0.4	4.0 ± 0.6	0.79
	Width of microconidia: mean ± sd	3.2 ± 0.4	3.2 ± 0.5	0.64
	Microconida L/W ratio	1.3 ± 0.1	1.2 ± 0.1	0.20
	Length of macroconidia: mean ± sd	31.9 ± 6.9	39.5 ± 7.5	0.42
	Width of macroconidia: mean ± sd	6.4 ± 1.8	8.0 ± 1.5	0.84
Clinical data	Clinical manifestation—onychomycosis : tinea pedis : tinea corporis	43 : 22 : 5	1 : 4 : 34	**<0.01**
	Age: range (median)	3–82 (57)	4–69 (27)	**<0.01**
	men : women	37 : 33	14 : 25	0.29
MAT gene determination	*MAT1-1-1* : *MAT1-2-1*	0 : 70	14 : 25	**<0.01**

^1^The characters were scored in 109 Czech clinical isolates or subset of strains.

#### Species vs. clinical data

Infection due to *T. interdigitale* manifested as tinea pedis (22/70 isolates) and onychomycosis (43/70 isolates) and less commonly as tinea corporis (5/70 isolates). *Trichophyton mentagrophytes* isolates were mostly isolated as a cause of tinea corporis (34/39) and less commonly as tinea pedis (4/39) and onychomycosis (1/39).

We found a significant association between species and the clinical manifestations of infection. While infection by*T. mentagrophytes* was not associated with any specific clinical manifestation, tinea pedis and onychomycosis were positively correlated with *T. interdigitale* (*p* < 0.01).

We also found a significant association between the age of patients and species. While the median age of patients infected by *T. mentagrophytes* was 27 years, the median age of patients infected by *T. interdigitale* was 57 years. As a result, dermatophytosis due to *T. interdigitale* was positively correlated with a higher patient age (*p* < 0.01). There was no association between the sex of the patients and species (*P* = 0.29).

#### Species vs. morphological characters

No significant differences in growth after 7 days at 25 °C were observed between *T. interdigitale* and *T. mentagrophytes* (*P* = 0.83). Strains belonging to *T. interdigitale* had significantly slower (*P* < 0.01) growth after 7 days at 37 °C (15 mm on average) than strains belonging to *T. mentagrophytes* (22 mm on average). We found no associations between species and phenotypic traits such as obverse color, reverse color, or colony texture (*P* = 0.12, *P* = 0.60, and*P* = 0.12).

Formation of spiral hyphae occurred significantly more frequently in *T. interdigitale* isolates in comparison with *T. mentagrophytes* isolates (*P* = 0.01). Other morphological features such as presence/absence of macroconidia, length of microconidia, width of microconidia, and length/width (L/W ratio) of microconidia were not significantly associated with species identification (*P* = 0.08, *P* = 0.79, *P* = 0.64, *P* = 0.20, respectively).

## Discussion

Controversy over the definition of species boundaries has long accompanied *T. mentagrophytes* and *T. interdigitale*, and this problem has not been resolved in the molecular era. Extensive interest in this complex is demonstrated by numerous epidemiological or taxonomic papers that question the monophyly of these taxa based on sequence data (Table 3). Unclear species definitions and unstable taxonomy have important consequences for practice when species-level pathogen identification is essential, and this controversy also limits the comparability of recent and past epidemiological data. An increasing number of authors, although using molecular data for species identification, prefer to use only the designation “*T. mentagrophytes* complex” and “*T. mentagrophytes/T. interdigitale*“, or to arbitrarily designate isolates from nails and feet as *T. interdigitale* and those from other body parts as *T. mentagrophytes*, thus ignoring sequences generated by themselves due to ambiguous signals or nontrivial interpretation.^42–45^

**Table 3.** Arguments supporting and opposing the simultaneous recognition of *T. interdigitale* and *T. mentagrophytes*

Arguments	Conclusions	References
Monophyly: multilocus phylogeny or phylogenomic data	TI and TM are not monophyletic	ITS + actin + *tubb* (Beguin et al.^9^); ITS + LSU + *tubb* (Pchelin et al.^64^); ITS + LSU + *tubb* + 60S L10 (de Hoog et al.^10^); ITS+ LSU + *tubb* (Suh et al.^23^); ITS + *tef1-α* (Tang et al.^12^); ITS + *tef1-α* + *tubb* (this study); phylogenomic data: Pchelin et al.^59^; Singh et al.^65^
Unique morphology (strains identified using molecular methods)	TI and TM cannot be reliably differentiated in practice (no strong features without significant overlap)	Heidemann et al.^4^; Dhib et al.^22^; Frías-De-León et al.^45^; Tang et al.^12^; this study
Unique clinical manifestation	TI is more frequently associated with onychomycosis and tinea pedis compared with TM	Heidemann et al.^4^; Dhib et al.^22^; Pchelin et al.^59^; Taghipour et al ^17^; Klinger et al.^3^; this study
Source of infection	TI is almost exclusively anthropophilic and TM is predominantly zoophilic	Heidemann et al.^4^; Nenoff et al.^19^; Taghipour et al.^17^; Klinger et al.^3^
Differentiation of TI and TM is important for treatment choice	Treatment guidelines for specific clinical units (tinea pedis, onychomycosis, etc.) and antifungal susceptibility testing are superior to species identification	See Discussion
Availability of simple and reliable molecular identification techniques for clinical practice	Identification relies on ITS genotyping (time-consuming and requires expertise); MALDI-TOF MS does not reliably distinguish TI and TM	Klinger et al.^3^; Uhrlaß et al.^16^; Suh et al.^23^; Normand et al.^62^; Tang et al.^61^

MALDI-TOF MS, Matrix-assisted laser desorption ionization-time of flight mass spectrometry; TI, *T. interdigitale*; TM, *T. mentagrophytes*.

In this study, we performed multigene phylogenetic analysis on 129 strains, 110 of which were collected from Czech patients, and species identity was defined according to the resulting tree. Correlations between assignment to *T. mentagrophytes* or *T. interdigitale* lineage, and phenotypic and clinical characteristics were investigated. We also summarize the advantages and disadvantages of preserving or merging species based on the studies carried out thus far (Table 3).

### Phylogenetic definition of species within *T. mentagrophytes* complex

Similar to previous studies, we confirmed that *T. mentagrophytes* is paraphyletic with respect to *T. interdigitale* when analyzing isolates using multiple-gene phylogeny or phylogenomic data (Table 3). The position of *T. indotineae* with respect to *T. mentagrophytes* and *T. interdigitale* is poorly resolved. Although it is located in basal position to these species in the combined best scoring ML tree (Fig. 1), the statistical support is very low. In the significant portion of ML trees, it was located in the sister position to *T. interdigitale* or within *T. mentagrophytes* lineages. *Trichophyton indotineae* shows only two unique substitution in the alignment of three loci (both in ITS region) differentiating it from both *T. mentagrophytes* and *T. interdigitale*. Other variable positions are either substitutions shared with one of the mentioned species or indels. When only substitutions are used for the construction of haplotype network, it is located between *T. mentagrophytes* and *T. interdigitale* strains (Fig. 2).

We demonstrated that the position of some isolates fluctuates significantly between the single-gene phylogenies, and not only between different clades of the *T. mentagrophytes* lineage, but also to a limited extent between *T. interdigitale, T. indotineae*, and *T. mentagrophytes* lineages. Therefore, considering *T. interdigitale* and *T. indotineae* only as a clonal offshoot of *T. mentagrophytes*^12,46^ is probably an oversimplification of the real situation; there is still some a gene flow or a significant shared ancestral polymorphism between these species. On the other hand, it is certainly true that *T. interdigitale* exhibits significant signs of clonality when using MLST approach while *T. mentagrophytes* is genetically diverse.

In practical terms, some isolates identified as *T. interdigitale* based on one gene would be identified as *T. mentagrophytes* based on another gene and vice versa. This complicates identification in practice and prevents species identification in some isolates. This fact also diminishes the importance of ITS region genotyping in the *T. mentagrophytes* lineage, which is increasingly used to characterize isolates of the *T. mentagrophytes* complex.^3,16,25,47^ It is unlikely that particular ITS genotypes of the *T. mentagrophytes* lineage will exhibit significantly different and stable biological features in the long term, as the differences between strains are diminished by recombination between sublineages. From a practical point of view, it may be appropriate to recognize ITS genotypes representing *T. interdigitale* and ITS genotype VIII of *T. mentagrophytes* (*T. indotineae*), which are associated either with a specific clinical manifestation or a more frequent occurrence of resistance.^3,48^ In this study, we identified several additional ITS genotypes (Table 1) that have not been reported in the literature,^16^ but based on the above reasons, we do not continue in their numbering.

Similar to this study, significant incongruence between single-gene genealogies and/or inability to satisfactorily identify some isolates when using multiple genes has been reported for some dermatophytes.^49–51^These problems have been described for instance among the two main populations of *Trichophyton erinacei*,^50^ and between sister species *Trichophyton tonsurans* and *Trichophyton equinum*.^49^ These observations were interpreted as ongoing speciation/incomplete divergence of species. Discordance of gene trees from the species tree due to ancestral polymorphism (incomplete lineage sorting) is a common problem across eucaryotic species and is more frequent in the evolutionary young species.^52,53^ From this perspective, it is possible that the markers selected in this study (in terms of their quantity or discriminatory power) may not be sufficient to separate lineages of *T. interdigitale* and *T. mentagrophytes* due to a high level of shared ancestral polymorphism.

### Intraspecific genetic variability

Only several *Trichophyton* species are believed to retain their ability to reproduce sexually, e.g., *T. mentagrophytes, T. africanum, T. simii, T. benhamiae* var. *benhamiae*, and *T. erinacei*.^50^,^51,54^ In these species, higher level of intraspecific genetic and phenotypic variability generated by the sexual reproduction is found. Among mentioned species, a MLST dataset comparable to that from the present study in terms of isolate number is only available for *T. erinacei* (71 isolates characterized by DNA sequences from four loci).^50^*Trichophyton erinacei* is comparable to *T. mentagrophytes* complex in several aspects. An ongoing process of speciation was seen in *T. erinacei* into two lineages, one specific mainly to *Atelerix* and second to *Erinaceus* hedgehogs. Although slight differences in the size of conidia and antifungal susceptibility patterns were observed among these population, they were not completely genetically separated and the differences were evaluated as not large enough to recognize them as separate taxonomic entities.^50^ The obvious analogy between *T. erinacei* and *T. mentagrophytes* encourages a further comparison of these two species in terms of intraspecific genetic variability. In the *T. erinacei* MLST datased published by Čmoková et al., a maximum sequence dissimilarity between isolates of *T. erinacei* is 1.4%. 1.7%, 0%, and 2% based on the ITS, *tef1-α, tubb*, and *gapdh* loci, respectively.^50^ While in our present dataset, the maximum sequence dissimilarity between isolates is 1.2%, 2%, and 1.6% based on the ITS, *tef1-α* and *tubb* loci, respectively. In this comparison, the variability within the broadly defined *T. mentagrophytes* is therefore comparable to *T. erinacei*. In other words, the inclusion of *T. interdigitale* and *T. indotineae* in *T. mentagrophytes* would not result in creation of a disproportionately large species. Similar comparison, published recently for 37 species across genus *Aspergillus* showed that intraspecific dissimilarites in the common taxonomic markers often reached up to 4%.^55,56^ Although these species have a different ecology, it turns out that the intraspecific variability in species that have a cryptic or unknown sexual cycle is greater than previously thought.

### Phenotypic definition

Our data showed that reliable differentiation of *T. interdigitale* and *T. mentagrophytes* was practically impossible using classical morphological features. Although we observed that isolates of *T. interdigitale* grew more slowly at 37 °C (a statistically significant result, but with an important overlap in the range of values) and more frequently produced spiral hyphae, these features are not reliable for identification in practice. No significant differences were found in the other characters that are routinely used for identification, such as colony diameter at 25 °C, colony color and texture, dimensions of conidia or production of macroconidia.

Our conclusions were in agreement with previous studies that examined relationships between genotype and phenotype and did not find any strong morphological features that were useful for routine differentiation (Table 3). Minor differences have been found for some features, such as colony reverse colour, colony texture and a keratin azure test,^4,12^ but these features are rather unstable and cannot be considered diagnostic and used for identification in practice.

In our study, we did not collect enough strains with genotype corresponding to the recently described *T. indotineae*. For that reason, statistical evaluation of characters and comparison with *T. interdigitale* and *T. mentagrophytes* could not be performed. It is, however, clear from available studies that the phenotypic features useful for differentiation in practice are missing.^12,18^ Although some characters appear to be statistically significantly different among mentioned species, there is considerable variability and overlap between all characters examined.

### Clinical and ecological definition

Onychomycosis and tinea pedis were more frequently associated with *T. interdigitale* compared with *T. mentagrophytes*. This finding is in agreement with several previous studies (Table 3). In our set of strains, all cases of onychomycosis were caused by *T. interdigitale* except for one case that affected toenails and was caused by *T. mentagrophytes*. The predominance of *T. interdigitale* over *T. mentagrophytes* as a cause of tinea pedis was much less significant, and we observed a ratio of 22:4 (84.6%) in favor of *T. interdigitale*. This situation clearly demonstrates that an arbitrary designation of isolates from nails and feet as *T. interdigitale* and designation of other isolates as *T. mentagrophytes*^44^ is an excessive oversimplification that leads to significant inaccuracies.

Patients infected with *T. interdigitale* in this study also had a significantly higher age than patients infected with *T. mentagrophytes*. This finding is, however, of secondary significance given that the average age of patients with onychomycosis is significantly higher than those with tinea corporis or tinea pedis.^57^

*Trichophyton interdigitale* and *T. mentagrophytes* are said to cause infections with different levels of inflammation.^3,7^ Leaving aside the fact that this criterion is partly subjective, Klinger *et al*. demonstrated that *T. mentagrophytes* isolates more often caused moderate to high inflammatory infections compared to *T. interdigitale*.^3^ On the other hand, 26 out of 45 *T. mentagrophytes* strains (those where information about inflammation was available) caused infections without inflammation or with low inflammation. In our strains, we did not observe any significant differences in the level of inflammation between tinea corporis and tinea pedis cases caused by *T. interdigitale* and *T. mentagrophytes*. Additionally, as observed by Tang et al., *T. interdigitale* strains could be isolated from infection sites that were typical for zoophilic species, such as the face and scalp.^12^ This fact further limits the possibility of making a species identification based on infection site.

Another often mentioned argument is that *T. interdigitale* isolates are mostly of human origin, while *T. mentagrophytes* isolates are of animal origin and their differentiation has consequences for tracking the source of infection and initiating preventive measures.^58^ This statement, although valid for *T. interdigitale* isolates from onychomycosis cases, is usually poorly substantiated by data from glabrous skin infections. Additionally, there is a lack of molecular data from animal isolates worldwide. For example, cat, dog, rabbit, and guinea pig isolates with a *T. interdigitale* genotype were published by Tang et al., documenting that the ecology of these “species” is not as distinct as previously thought.^12^ In addition, there are intermediate genotypes between *T. interdigitale* and *T. mentagrophytes* that more frequently originate from animals.^4,12,16,59^ Similarly, *T. indotineae*, which is usually designated as anthropophilic species, occurs in animals. It has been detected in calves in Egypt, dogs in India and unspecified animals in Poland.^20,21^ These findings indicate that zoonotic transmission must be also considered in *T. indotineae* and the sporadic occurrence in animals may only reflect the lack of studies in the veterinary field in the affected countries. In this study, we also report the first isolate with genotype of *T. indotineae* from the Czech Republic. This clinical strain CCF 6599, isolated in 2016, was susceptible to terbinafine (MIC 0.016 mg/l; EUCAST E.Def 11.0).^60^

### Concluding remarks

In this study, we showed that the separation of *T. interdigitale* and *T. mentagrophytes* is incomplete. These species are not monophyletic and their phenotypic divergence is poor or absent. From the point of view of classical taxonomy, we have very few arguments for preserving both species names. The main reason for differentiation of these “species” is that the source of infections and clinical manifestation is relatively unique for some genotypes associated with *T. interdigitale* (ITS genotypes I, II, X, XI, and XII) and *T. indotineae* (ITS genotype VIII).

The currently recognized species within *T. mentagrophytes* complex do not meet the usual taxonomic criteria and from the point of view of routine practice, it is essential that these names cannot be applied to all isolates of the complex due to the presence of intermediate genetic and phenotypic forms. The differentiation of these “species” using MALDI-TOF mass spectrometry, a widely used method in clinical practice, usually fails.^23,61^ This method seems to be effective only in distinguishing *T. indotineae* from the *T. interdigitale*/*T. mentagrophytes* isolates.^61,62^ Additionally, the clinical significance of the names *T. mentagrophytes, T. interdigitale* or *T. indotineae* is not essential in the choice of treatment, which is preferably guided by the recommendations for a given clinical unit combined with knowledge of the local situation about the level of resistance. The occurrence of resistance in individual isolates should be confirmed by antifungal susceptibility testing or mutation analysis, as resistance is not an intrinsic characteristic of any species or genotype in the complex, but it is usually associated with the ITS genotype VIII.^48,63^ In other words, neither the isolation of a strain from a particular location on the body, information on the level of inflammation, or ITS genotype data provide exact information about the identity, resistance and origin of an isolate. There are patterns in the geographical distribution of ITS genotypes and in their predominant association with certain clinical types.^3,17^ However, these trends are far from exhibiting the accuracy on which diagnoses should rely, and their clinical relevance is limited.

**Figure 4.**
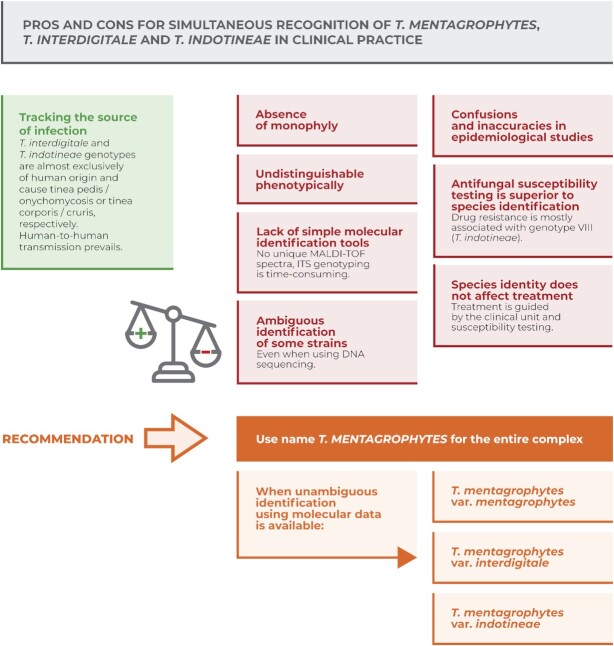
Summary of arguments supporting and opposing the simultaneous recognition of *T. mentagrophytes, T. interdigitale*, and *T. indotineae* within the *T. mentagrophytes* complex, and taxonomic recommendations on naming taxa.

Based on these arguments summarized in Fig. 4, we recommend using the name *T. mentagrophytes* for all isolates in the complex, including *T. interdigitale* and *T. indotineae*. When unambiguous molecular identification of *T. interdigitale* and *T. indotineae* is possible, we recommend optionally using a variety rank: *T. mentagrophytes* var. *interdigitale* and *T. mentagrophytes* var. *indotineae*. We believe that this practice will contribute to the establishment of a broadly understandable taxonomy of this complex and will avoid further confusions and simplify identification in practice.

## Taxonomy

***Trichophyton mentagrophytes* var. *indotineae* (R. Kano et al.) Švarcová, M. Kolařík and Hubka, stat. nov**. The MycoBank deposit number is MB847789.

*Basionym: Trichophyton indotineae* R. Kano et al., Mycopathologia 185: 957. 2020. MycoBank MB833488.

For species description, see Kano et al.^18^ The holotype specimen of *Trichophyton indotineae* is preserved in the herbarium of the Medical Mycology Research Center (MMRC), Chiba University, Japan (IFM 66168). Ex-type strain: NUBS 19006 = CBS 146623 = NCCPF IL4163 = CCF 6597.

## Supplementary Material

myad042_Supplemental_Files
