# Supplementary material for: Defining the relationship between phylogeny, clinical manifestation, and phenotype for Trichophyton mentagrophytes/interdigitale complex; a literature review and taxonomic recommendations
Source: Med Mycol. 2023 Apr 17;61(5):myad042. doi: 10.1093/mmy/myad042 (PMC10148955; doi:10.1093/mmy/myad042)
Supplement: myad042_Supplemental_Files [file myad042_supplemental_files.zip › mm-2022-0246-File010.docx]

|  | | | |
| --- | --- | --- | --- |
| **Table S2.** Characteristics of alignments, partition-merging results and best substitution model for each partition according to Bayesian information criterion | | | |
| **Alignment** | **Length (bp)** | **Variable position** | **Partitioning scheme (substitution model)** |
| ITS, *tef1-α* & *tubb* | 1644 | 74 | Seven partitions: ITS1 & ITS2 (TrN+G); 5.8S (K80); 1^st^ codon positions of *tef1-α* (F81+I); 1^st^ codon positions of *tubb*; 2^nd^ codon positions of *tubb* & *tef1-α* (JC); 3^rd^ codon positions of *tubb* & *tef1-α* (HKY+I); introns of *tubb* & *tef1-α* (K80+I+G) |
| ITS | 594 | 22 | Two partitions: ITS1 & ITS2 (TrN+G); 5.8S (K80) |
| *tef1-α* | 605 | 36 | Three partitions: 1^st^ & 2^nd^ codon positions (JC); 3^rd^ codon positions (HKY+I); introns (K80) |
| *tubb* | 445 | 16 | Three partitions: 1^st^ & 2^nd^ codon positions (JC); 3^rd^ codon positions (HKY); introns (K80+G) |
